# Contemporary Techniques for Femoral and Radial Arterial Access in the Cardiac Catheterization Laboratory

**DOI:** 10.31083/j.rcm2309316

**Published:** 2022-09-14

**Authors:** James D. Gladden, Rajiv Gulati, Yader Sandoval

**Affiliations:** ^1^Department of Cardiovascular Diseases, Mayo Clinic, Rochester, MN 55905, USA

**Keywords:** vascular access, catheterization laboratory

## Abstract

Safe and efficient arterial access is critical for optimal patient outcomes and procedural success in the cardiac catheterization laboratory. Because of the lower risk for vascular and bleeding complications, as well as patient comfort, transradial access has become the predominant approach for diagnostic coronary angiography and percutaneous coronary intervention. Transfemoral access, however, is still required for selected complex percutaneous coronary interventions, mechanical circulatory support, and structural heart procedures. The use of adjunctive technology and techniques such as ultrasound guidance and micropuncture can be combined with fluoroscopy and palpation to improve outcomes associated with vascular access. The importance of optimal access techniques has augmented due to increasing volume of structural heart and mechanical circulatory support procedures requiring large bore sheaths. In this document we review the contemporary techniques for femoral and radial access in the cardiac catheterization laboratory.

## 1. Introduction

First described in 1953 by Ivar Seldinger [1], percutaneous arterial access via the common femoral artery (CFA) was historically the most common method of vascular access for coronary catheterization procedures. Following extensive data, including randomized trials showing that radial access is superior to femoral access with respect to bleeding and vascular complications [2, 3, 4, 5, 6, 7, 8, 9, 10], transradial access has become the predominant route for coronary angiography and percutaneous coronary interventions. Obtaining safe vascular access in an efficient and reproducible manner with low complication rates is a priority in the cardiac catheterization laboratory. Here, we review the current state of the art practice for femoral and radial arterial access, as well as discuss large bore access given its increasing use related to mechanical circulatory support and structural procedures.

## 2. Femoral Arterial Access

**Fig. 1.**
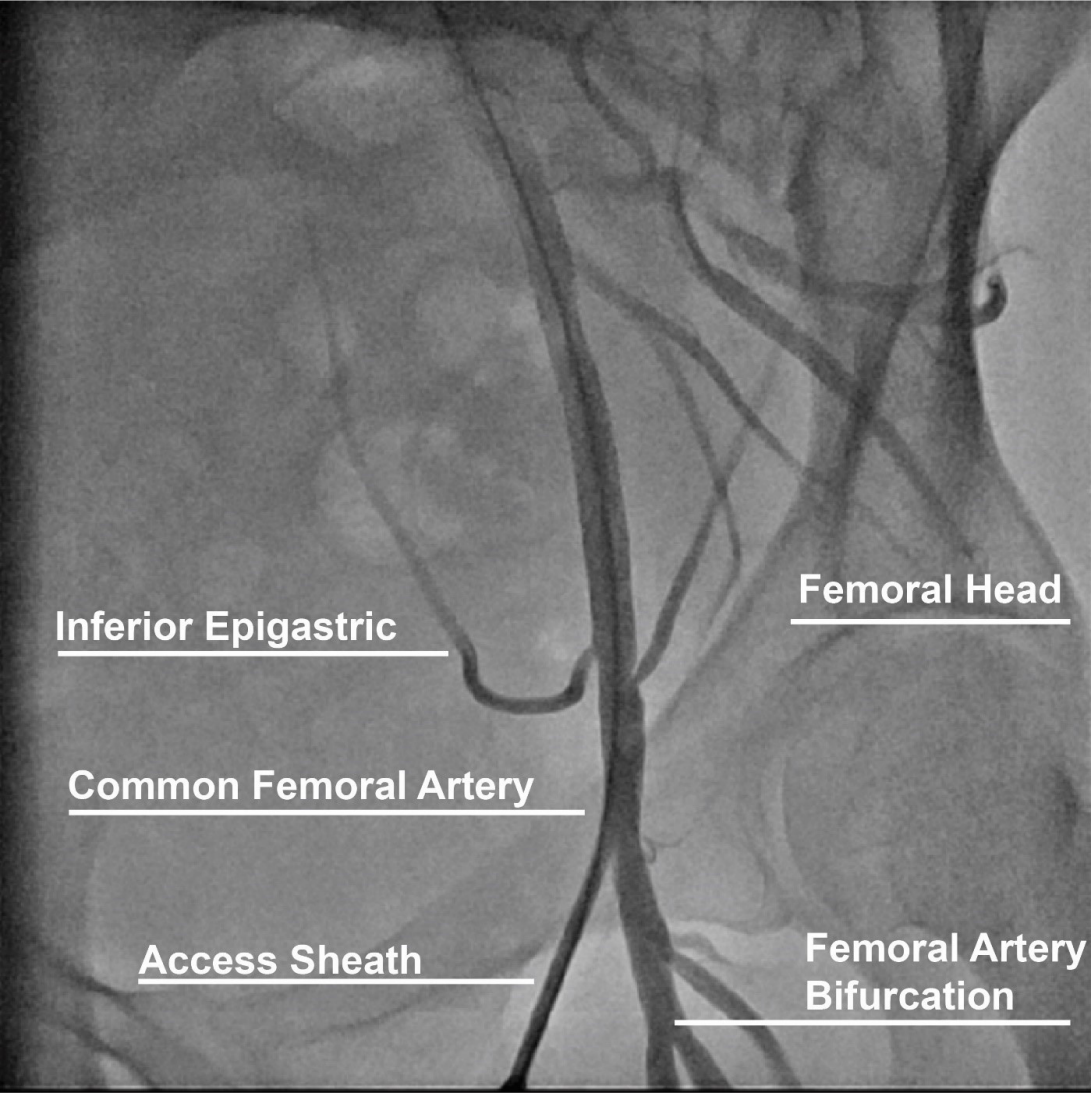
**Anatomical landmarks for femoral access**.

The CFA originates from the external iliac artery and crosses under the inguinal ligament and branches into the superficial (SFA) and profunda (PFA) femoral arteries distally (Fig. 1) [11]. The large caliber and ability to compress the artery over the femoral head of the CFA make this arterial anatomical site the preferred for procedures requiring femoral access. Successful CFA access is established when the sheath is inserted into the CFA above the bifurcation of the SFA and PFA and below the inferior epigastric branch in an area compressible against the femoral head. Anatomical landmarks to maximize CFA access include finding the anterior superior iliac spine laterally and the symphysis pubis as a landmark for the inguinal ligament and obtaining access 2–3 centimeters below the midpoint between these landmarks. However, anatomical variations such as high bifurcation and obesity diminish the reproducibility and accuracy of this technique [12]. Low access increases the risk for bleeding and hematoma due to lack of a compressible site, and pseudoaneurysm [13, 14]. High access (above the inguinal ligament) increases the risk of a retroperitoneal bleed, which is associated with a 3-fold increase in mortality and a 5-fold increase in adverse outcomes [15]. Other predictors of retroperitoneal bleeding include low body weight, female sex, larger sheath size, and use of glycoprotein IIb/IIIa inhibitors [16, 17]. Therefore, adjunctive techniques to accurately access the CFA such as fluoroscopy and ultrasound have been implemented to contemporary femoral access techniques and will be discussed in the following sections.

## 3. Ultrasound Guidance for Femoral Arterial Access

Ultrasound guidance for femoral access provides real-time anatomical information [18]. Randomized data and meta-analyses show that ultrasound-guided is associated with a lower number of attempts, higher first past success rate, lower risk of venipuncture, lower time to sheath insertion, and improved CFA access in those with high bifurcation [19, 20]. While radial access is the preferred route for most coronary procedures in contemporary practice, femoral access is required for mechanical circulatory support and structural procedures, as well as often used in patients with prior coronary artery bypass graft surgery [21], cases with poor radial or ulnar access in which femoral is favored or crossover is needed, or in selected complex percutaneous coronary procedures where larger bore sheaths and/or or longer sheaths for support are needed such in coronary total occlusion PCI where dual access is recommended [22]. Two-dimensional ultrasound in short and long-axis allow visualization of the CFA bifurcation into the SFA and PFA inferiorly [23]. By increasing ultrasound depth, the femoral head can also be observed and be used to guide needle access over such. There is robust evidence supporting the superiority of ultrasound guidance for femoral artery access [19]. A meta-analysis of 4 trials addressing 1422 subjects demonstrated that ultrasound guidance was associated with fewer life-threatening vascular complications and improvement in first attempt access success as compared to palpation [20]. In FAUST, the largest randomized trial comparing ultrasound vs. fluoroscopy, the use of ultrasound reduced the number of access attempts (3.0 ± 3.2 vs. 1.3 ± 0.9; *p *< 0.00001) and first attempt success (46% vs. 83%; *p *< 0.000001) [19]. Large hematomas (>5 cm) were also less likely using ultrasound as compared to palpation (0.6% vs. 2.2%, *p* = 0.03). Similar findings have been observed for femoral venous access in patients undergoing electrophysiology procedures [24, 25], as well as in studies addressing ultrasound-guided vascular access for peripheral interventions [26]. In patients with a high CFA bifurcation, SFA access may be an alternative with data from a small trial showing similar pseudoaneurysm rates as CFA access when using closure devices [27].

## 4. Contemporary Femoral Arterial Access 

**Fig. 2.**
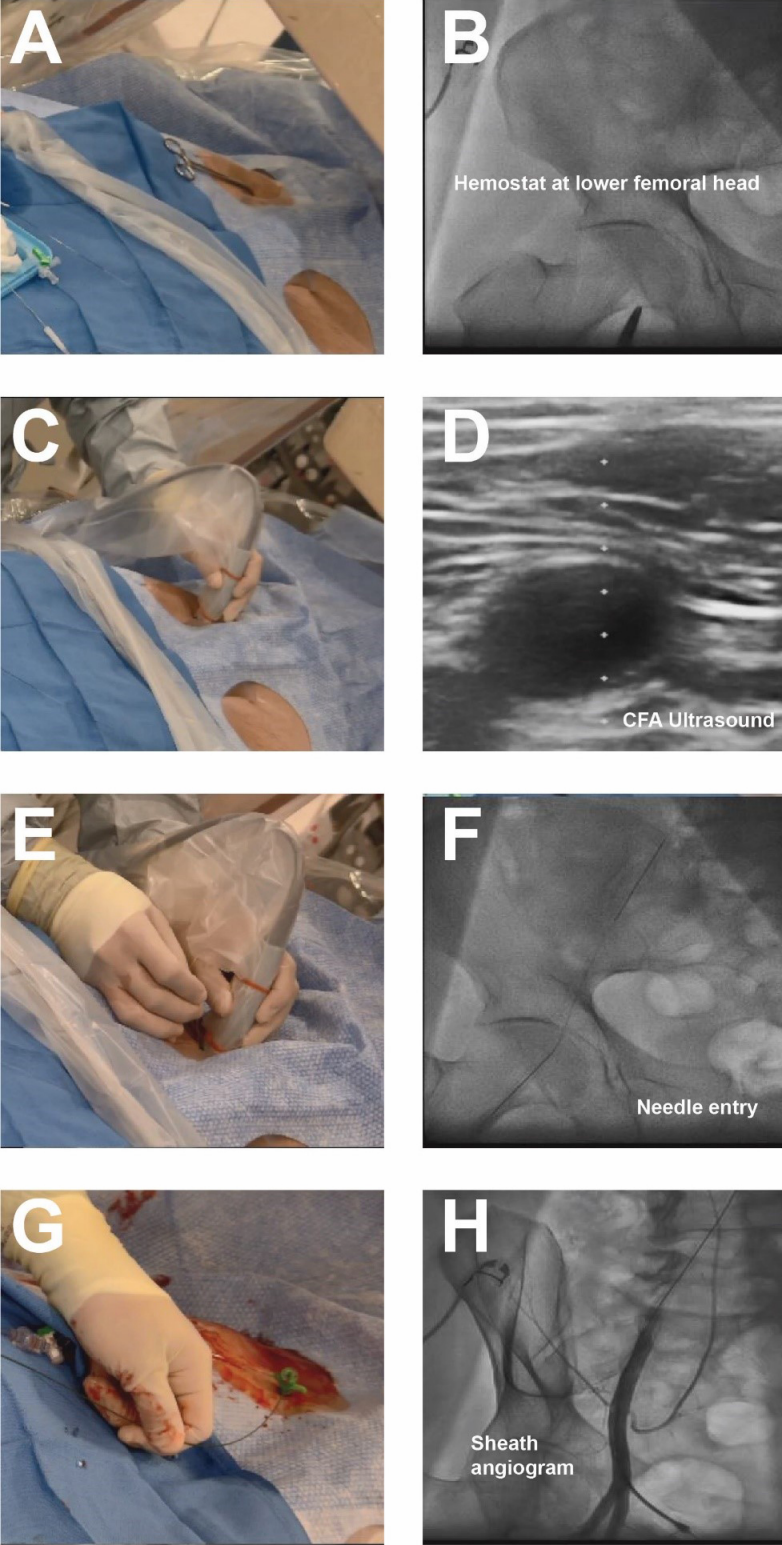
**Contemporary femoral access techniques**. (A) Placement of hemostat to mark lower edge of femoral head. (B) Fluoroscopy of hemostat. (C,D) Ultrasound of CFA. (E,F) Needle placement and fluoroscopy of needle entry into CFA. (G,H) Placement of 6 french sheath and femoral angiogram to confirm anatomical location, note presence of wire in sheath.

**Fig. 3.**
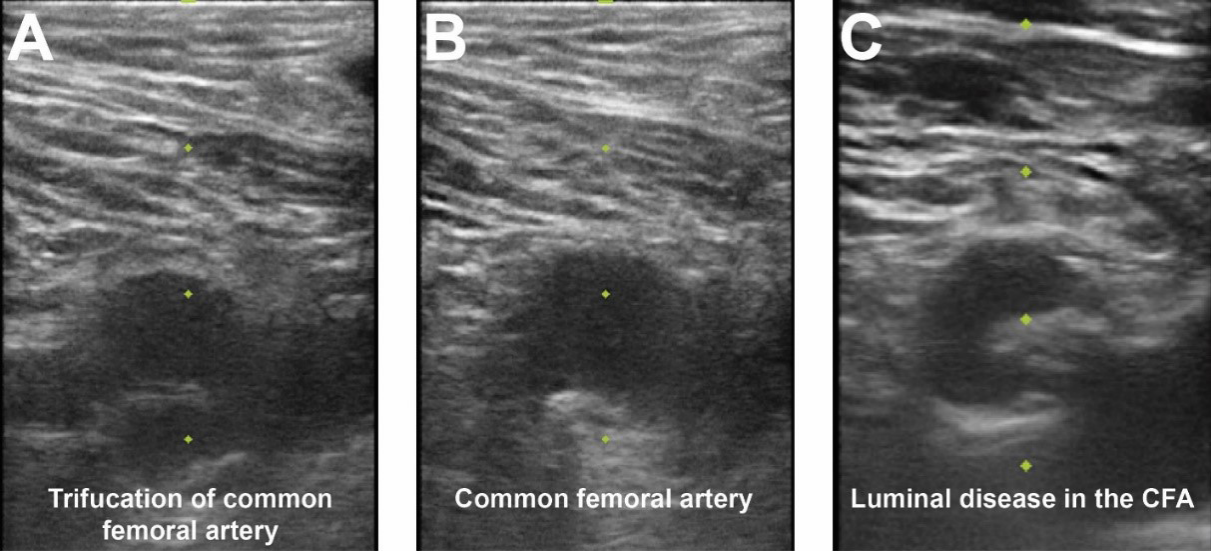
**Femoral access ultrasound**. (A) Femoral artery ultrasound shows a trifurcation of the CFA and femoral vein to image right. (B) Superior to the CFA bifurcation is seen in an area amenable for vascular access. (C) CFA ultrasound noting significant luminal atherescolosis.

**Fig. 4.**
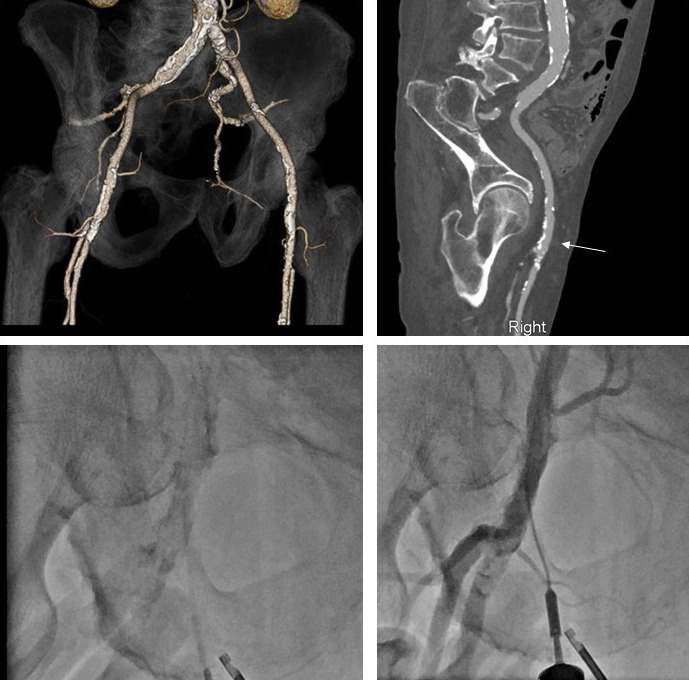
**Computed tomography, fluoroscopy, and femoral angiography for femoral access**. (Top left panel) CTA obtained prior to TAVR notes extensive calcifications of the left and right iliofemoral system. (Top right panel) Muti-planer reconstruction of the right iliofemoral system with area of planned vascular access notated with white arrow. This area was chosen due to lack of anterior calcification. (Bottom right panel) Fluoroscopic evidence of dense calcification of the right CFA. (Bottom right panel) Femoral angiogram following TAVR procedure and deployment of 2 Proglide sutures. This was achieved by passing a micropuncture sheath over the guidewire prior to finally locking the sutures. Femoral angiogram noted patent femoral vessels without change from baseline, vascular no complications and adequate hemostasis.

**Fig. 5.**
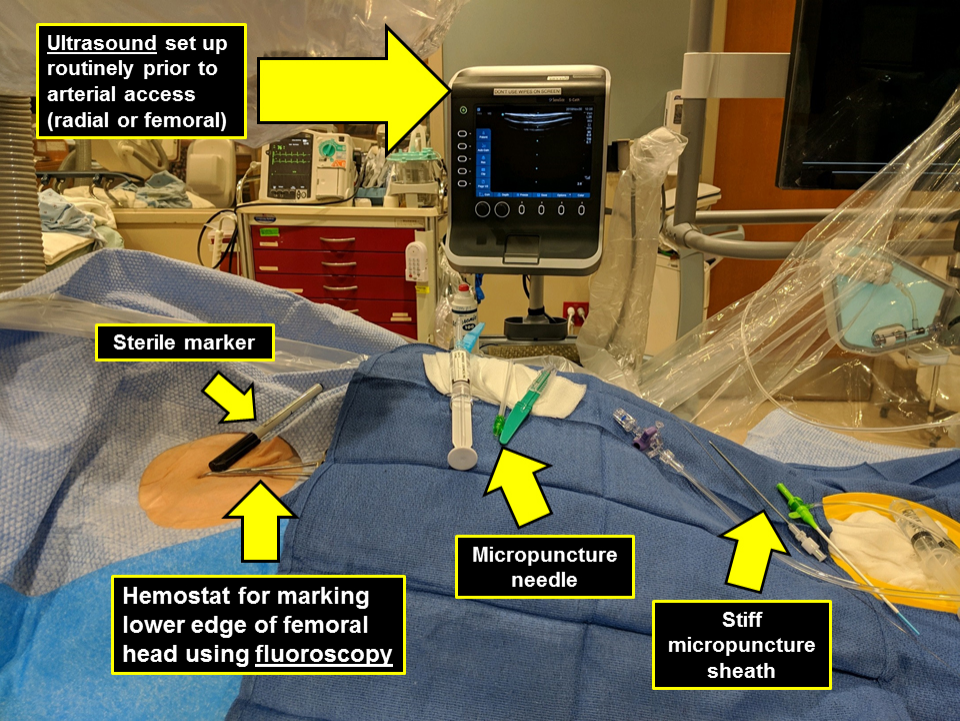
**Catheterization laboratory setup for femoral access**.

Contemporary femoral access should utilize all available techniques and adjunctive information available to reproducibly achieve vascular access with the lowest complication rate possible [18]. With the patient supine on the catheterization table, anatomic landmarks should be assessed. In patients with a large body mass index, retraction of the pannus can facilitate vascular access as well as hemostasis and closure. Placement of a radiopaque marker (hemostat) should be utilized in conjunction with fluoroscopy to assess the lower edge of the femoral head (Fig. 2). This location can be marked with a sterile pen and may help avoid high puncture. Ultrasound should then be performed to assess the ideal entry point for CFA access. The ultrasound probe can initially be placed perpendicular to the patient at the location of the lower edge of the femoral head as previously identified. Prior to attempting access the operator should assess the femoral artery anatomy including the location of the CFA bifurcation into SFA and PFA and evaluate for any major branch vessel, areas with severe calcification or obstructive peripheral arterial disease that should be avoided (Figs. 3,4). Longitudinal assessment of the CFA as it dives into the pelvis can also help the operator avoid high access, as well as visualization of the femoral head. Once the ideal target area of vascular access has been located with the ultrasound probe, careful attention should be given to any manipulation of the ultrasound probe. The ultrasound probe should be static and maintained straight without any tilting or angulation. Skin puncture with the access needle should occur 1–2 cm distal to the probe while aligned with the center marker on the probe and approximately 30–45 degrees. Steep angulations should be avoided as they can contribute to sheath or wire kinking. Following needle entry into the CFA, a J-tipped or micropuncture wire is introduced. Routine fluoroscopic assessment of needle entry location with the J-tipped/micropuncture wire in place (Fig. 2) should be performed to confirm that the needle entry is over the femoral head. If the needle entry is below or above the femoral head, this safety step allows for removal of the needle to reattempt access. If the needle entry site is satisfactory, then the wire can be advanced, however, tracking of the access wire should be pursued fluoroscopically when using the micropuncture wire as it can enter side-branches such as the inferior epigastric or deep circumflex iliac branches and cause perforations [28]. Following sheath insertion, femoral artery angiography [usually 30 degrees right anterior oblique (RAO) for right CFA access and 30 degrees for left CFA access] should be obtained to confirm safe access without complications, as well as help evaluate the anatomy and assess for tortuosity or peripheral arterial disease (Fig. 2). Occasionally additional angiographic projections may be required to confirm entry site. While femoral angiography can be performed through the micropuncture sheath, the latter provides limited opacification and has the risk for complications such as vessel dissection given that the injection is performed without assessment of hemodynamic waveform and the microcatheter sheath may be positioned against the vessel wall. Therefore, our suggested approach is that femoral angiography should preferably occur with the J-wire in place as such deflects the sheath from the vessel wall and maintains vessel control should complications occur. C-arm rotation can also be utilized to facilitate needle entry visualization. A standard setup for contemporary femoral access is shown in Fig. 5.

Vessel puncture can be obtained with either a standard 18G needle or a 21G micropuncture needle. The micropuncture needle is smaller in diameter (21-gauge) as compared to the traditional 18-gauge vascular access needle. The micropuncture technique allows the operator to withdraw the needle from an access attempt that resulted in a poor location with less hemostatic sequela due to its reduced diameter. Manual pressure can be held for a few minutes and another attempt can be made. This can be particularly helpful in obese patients, those that are anticoagulated, or in cases of large bore access where precise vascular entry point can be critical. The FEMORIS study was a single-center trial with 402 patients randomized to 18-gauge vs 21-gauge micropuncture access needle [29]. This study did not demonstrate a difference in the primary endpoint of composite bleeding, but the study was underpowered and terminated prematurely. However, lower bleeding rates were seen in the pre-specified subgroups such as women (17.4% vs. 5.8%; *p* = 0.05) and those with final sheath size ≤6 Fr (15.1% vs. 6.4%; *p* = 0.02) [29]. More data is needed comparing micropuncture with the traditional 18-gauge access needle. While there are advantages to the micropuncture approach, in patients with morbid obesity and/or vessel calcification, the micropuncture sheath can kink and make it challenging to advance the wire, for which reason, in some cases a standard 18G needle and a long J wire can be used under fluoroscopic and ultrasound guidance to provide more support for sheath insertion.

## 5. Radial Arterial Access 

Radial artery access for coronary angiography was first described in 1989 [30]. Radial access for coronary angiography has become the predominant route of arterial access for coronary angiography and PCI due to the reduced risk for vascular and bleeding complications as compared to femoral access, as well as easier patient recovery and ambulation [31]. A recent meta-analysis involving 31 trial and 30,096 patients noted that radial access was associated with a significant reduction in bleeding, vascular complications, and mortality compared to femoral access [3]. Multiple studies demonstrated less vascular complications especially in patients with ST-elevation myocardial infarction (STEMI) [5, 9, 32]. European and American guidelines recommend radial access as the preferred approach for coronary angiography and PCI when possible [33, 34]. Distal transradial access is an emerging technique that has been recently evaluated in randomized trials and has been discussed elsewhere [35, 36].

## 6. Ultrasound Guidance for Radial Arterial Access

Ultrasound permits precise assessment of vessel location as compared to palpation; it allows for assessment of the vessel size as well as for anatomical variations such as dual radial arteries, and permits evaluation of the ulnar artery in case the radial artery is small. Extensive data supports its use over palpation [37, 38]. The most recent meta-analysis of ultrasound guided radial artery access encompassed both adult and pediatric patients [38]. This study included 11 randomized controlled trial. In adults, compared with the control group, ultrasound guidance significantly improved first-attempt success rate (RR 1.4; 95% CI 1.28–1.64; *p *< 0.00001) with similar findings in pediatric patients. The largest randomized study of ultrasound for radial artery access was the RAUST trial [37], in which 698 patients were randomized to palpation vs. ultrasound guidance. First-attempt success, number of attempts, and time to access were superior with ultrasound guidance as compared to palpation.

## 7. Contemporary Radial Arterial Access 

With the patient supine on the catheterization table, the radial artery pulse should be assessed, however absence of radial artery pulse does not preclude radial access. Ultrasound can provide adjunctive information regarding radial artery size, presence of calcification, anomalies, and branching pattern. Performing Allen’s test to demonstrate patency of the ulnopalmar arch is not required prior to proceeding with radial access [39]. Radial access can be obtained using either a modified Seldinger (anterior wall puncture) or a Seldinger technique (through-and-through). Randomized data showsthat the Seldinger technique is superior to the modified Seldinger technique with respect to access and procedure time, number of attempts and first attempt access, as well as crossover [40]. Both techniques can be used in conjunction with ultrasound.

The ultrasound probe should be positioned proximal to the radial styloid process. The radial artery is a relatively superficial vessel and therefore we suggest that needle entry can be aligned and positioned next to the center of the probe, and once the vessel is centered on the ultrasound screen, then vessel puncture can be obtained with ease. Once the artery is entered, if using the through-and-through Seldinger technique, then the needle sheath is pulled until arterial pulsatile flow is observed at which time wiring is performed. Alternatively, if using the anterior modified Seldinger technique, coaxial needle positioning to the vessel lumen is essential to facilitate wiring, for which it is important to ensure that pulsatile blood flow is observed, and lower needle angulation can often help wiring. Coaxial positioning with the latter technique is essential as vascular complications such as dissection, spasm, or hematoma can occur if the wire is advanced into a subintimal track.

Anomalous radial artery anatomy has been reported to occur in in 13.8% of patients in one study of 1540 consecutive patients undergoing access-site angiography [41]. Among these cases, a high-bifurcating radial origin (7.0%) was the most common anomaly, followed radial loops, extreme radial artery tortuosity, and other miscellaneous anomalies. For these reasons some operators advocate for routine angiography following radial access to establish such diagnoses prior to additional instrumentation. Specialized equipment can be helpful in these cases with the usage of a steerable soft-tip guidewire or a hydrophilic wire. Additionally, navigation with a coronary wire and balloon-assisted tracking [42] can be helpful in cases with severe tortuosity or spasm. Distal transradial access in the anatomic snuffbox is increasingly used given emerging data suggesting low radial artery occlusion rates and operator comfort when using a left radial approach, as well as because of the development of dedicated compressive, hemostatic equipment [43, 44, 45]. Radial artery access complications are infrequent but can be associated with morbid consequences if not recognized early or treated. We have discussed the management of radial complications elsewhere [46].

## 8. Special Circumstances and Large Bore Access Considerations

Femoral access remains the predominant option for large bore arterial procedures. Slender sheaths and sheathless guides facilitate the ability to perform most procedures via the radial approach. Femoral access, however, is required for mechanical circulatory support, structural procedures such as transcatheter aortic valve replacement, and often used for chronic total occlusion (CTO) PCI in which 2 arterial access sites are needed [47]. There is emerging evidence of the safety and efficacy of bi-radial access for CTO interventions [48]. For non-emergent procedures, careful vascular access planning is a crucial component of large bore access. For TAVR it is common to obtain a CT angiogram of the vascular access options (Fig. 4). The location of vascular access can be chosen and then targeted during the case to precisely enter the CFA at the pre-planned location to decrease the risk of vascular complications (Fig. 4). Besides CT, other non-invasive imaging modalities that can inform vessel size, anatomy, and presence of peripheral arterial disease, include ultrasound, vascular non-contrast CT which allows vessel sizing and assessment of anatomy and tortuosity but not stenoses, and magnetic resonance imaging.

## 9. Conclusions

Radial access is the preferred approach to diagnostic coronary angiography and percutaneous coronary intervention. Femoral access, however, continues to be the primary option for large bore access, including for selected complex coronary interventions, transcatheter aortic valve replacement, and mechanical circulatory support. The routine use of ultrasound for any vascular access, as well as complementary use of fluoroscopy and angiography for femoral access can facilitate safe and efficient arterial access.
